# Long-term survivor of hepatocellular carcinoma treated with repeated carbon ion radiotherapy and transarterial chemoembolization: a case report

**DOI:** 10.1007/s12328-022-01642-4

**Published:** 2022-06-09

**Authors:** Takeru Ohtaka, Shintaro Shiba, Kei Shibuya, Shohei Okazaki, Yuhei Miyasaka, Kento Tomizawa, Masahiko Okamoto, Tatsuya Ohno

**Affiliations:** 1 Department of Radiation Oncology, Gunma University Graduate School of Medicine, 3-39-22, Showa-machi, Maebashi, Gunma 371-8511 Japan; 2 Gunma University Heavy Ion Medical Center, 3-39-22, Showa-machi, Maebashi, Gunma 371-8511 Japan

**Keywords:** Hepatocellular carcinoma, Carbon ion radiotherapy, Re-irradiation, Transarterial chemoembolization, Multidisciplinary treatment

## Abstract

Hepatocellular carcinoma (HCC) often recurs in the liver and requires multiple rounds of treatment. Thus, less-invasive multidisciplinary approaches are essential for preserving liver function, especially in elderly patients. Here, we report a case of an 86 year-old Japanese male patient with HCC who was successfully treated with repeated carbon ion radiotherapy (C-ion RT) and transarterial chemoembolization (TACE). The patient had alcoholic liver cirrhosis with a 60 mm HCC lesion and a satellite lesion in segment 6. The patient underwent initial C-ion RT but developed primary tumor recurrence (segment 6) and a new lesion (segment 2) 24 months later. The patient received TACE for each lesion, followed by an increased dose of C-ion RT for the recurrent primary tumor. Although the primary tumor lesion was well controlled, the patient subsequently developed new lesions, and TACE was repeated. The patient died of bacterial pneumonia 88 months after the initial treatment. His general condition and liver function were well preserved, and no severe adverse events were observed throughout the course of treatment. These results suggest that a less-invasive multidisciplinary approach involving repeated C-ion RT combined with TACE enables preservation of liver function, which may contribute to long-term survival in elderly patients with HCC.

## Introduction

Hepatocellular carcinoma (HCC) often develops from liver cirrhosis, which can be caused by hepatitis caused by viral infection, alcohol consumption, or non-alcoholic fatty liver disease. Recurrence of HCC is not uncommon. In addition, the age of HCC patients is increasing, with the average age at diagnosis in men and women being 67.8 and 71.2 years, respectively, in Japan [1]. As such, there is an urgent need for less invasive treatment options.

Surgical resection and liver transplantation are considered curative treatments for HCC. However, they are highly invasive, especially for aging patients, and are limited to a few patients due to tumor extension and poor hepatic function [2, 3]. Although radiofrequency ablation (RFA) is a less invasive procedure, it is not suitable for patients with large tumors or tumors in unfavorable anatomical locations [4]. Stereotactic body radiotherapy (SBRT) is also not suitable for patients with large HCCs [5]. Transarterial chemoembolization (TACE) can often be applied to patients whose treatments are difficult and can result in favorable local treatment effects. However, local recurrence is frequently observed with TACE alone [6]. TACE is also used to enhance the local effects of RFA and SBRT [7, 8].

Carbon ion radiotherapy (C-ion RT) is a promising radiotherapy modality that allows higher dose localization and relative biological effectiveness (RBE) than X-ray radiotherapy [9, 10]. Previous studies of dosimetric comparisons between C-ion RT and SBRT for HCC have shown that C-ion RT has better sparing of the surrounding normal liver, especially in large tumors [11, 12]. Due to these advantages of physical and biological aspects compared to X-ray RT, clinical results of C-ion RT for HCC have been reported to have a highly local effect with less severe toxicities, despite including large tumors [13]. C-ion RT is also well-tolerated in elderly and fragile patients with HCC [14, 15]. Here, we present a case of an elderly patient with a large HCC tumor treated with repeated C-ion RT and TACE.

## Case report

**Fig. 1:**
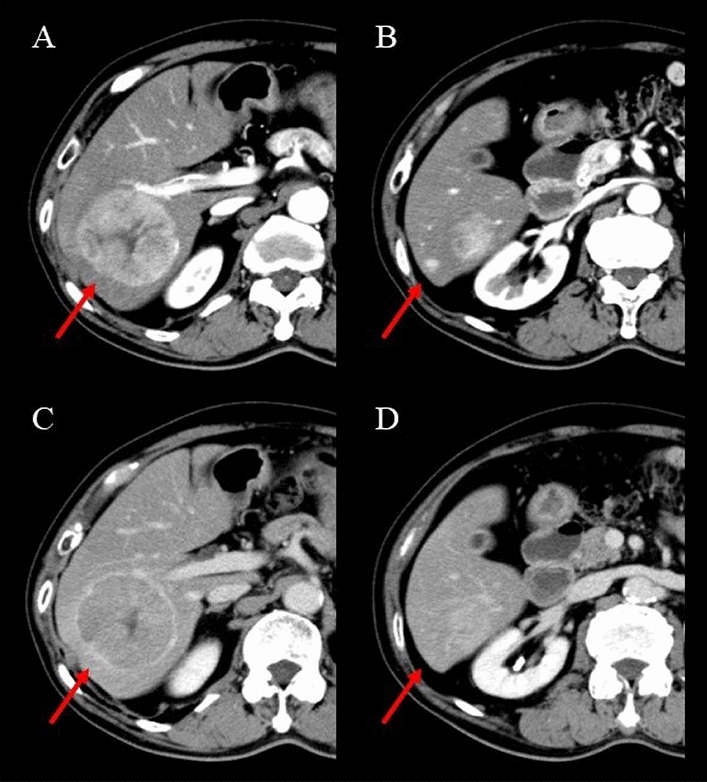
Contrast-enhanced CT at diagnosis. **A**, **B** Arterial phase CT, red arrows show the primary tumor (**A**) and the satellite nodule in segment 6 (**B**) with arterial enhancement. **C**, **D** Delayed phase CT, red arrows show washout of the primary tumor (**C**) and the satellite nodule (**D**)

An 86-year-old male Japanese patient with HCC was referred to the Gunma University Heavy Ion Medical Center. The patient had liver cirrhosis caused by heavy alcohol consumption. His Eastern Cooperative Oncology Group performance status was 1. The patient’s liver function status was Child–Pugh class A (score: 5) and modified albumin–bilirubin (mALBI) grade 1 (score: – 2.86). Laboratory tests results are summarized in Table 1. Total bilirubin, albumin, prothrombin time were all within normal ranges. Alpha-fetoprotein (3.3 IU/L; reference range 0–7.0 IU/L) and platelets (158,000 cells/μL; reference range 160,000–350,000 cells/μL) were also normal. At 15 min (ICG-R15), the indocyanine green retention rate was 17.5%. Contrast-enhanced computed tomography (CT) and gadolinium–ethoxybenzyl–diethylenetriamine pentaacetic acid (Gd–EOB–DTPA)-enhanced magnetic resonance imaging (MRI) detected a tumor (6.0 × 5.8 × 5.4 cm) and a satellite nodule in segment 6 of the liver, with early enhancement in the arterial phase and washout in the delayed phase (Fig. 1).

**Table 1:** Parameters of liver function before or after each treatment

	Before C-ion RT	24 months after C-ion RT (Before TACE)	27 months after initial C-ion RT (Before 2nd C-ion RT)	42 months after initial C-ion RT (Before 2nd TACE)	60 months after initial C-ion RT (After all treatment for HCC)
T-bil (mg/dL)	0.7	0.7	0.6	0.5	0.6
Alb (mg/dL)	4.2	4.0	3.8	3.6	3.9
PT ratio (%)	102	102	108	111	115
ICG-R15 (%)	17.5	N/A	14.2	N/A	N/A
Ascites	None	None	None	None	None
Hepatic encephalopathy	None	None	None	None	None
Child–Pugh class (score)	A (5)	A (5)	A (5)	A (5)	A (5)
mALBI grade (score)	1 ( – 2.86)	1 ( – 2.69)	2a ( – 2.56)	2a ( – 2.44)	1 ( – 2.65)

*T-Bil* total bilirubin (reference range 0.3–1.2), *Alb* albumin (reference range 3.9–5.0), *PT* prothrombin time (reference range 70–130), *ICG-R15* indocyanine green retention rate at 15 min, *mALBI* modified albumin–bilirubin

In addition, CT and MRI showed no ascites and no evidence of metastasis to the lymph nodes or distant organs. The patient was diagnosed with stage III (clinical T3aN0M0) HCC according to the 7th edition of the Union for International Cancer Control/American Joint Committee on Cancer TNM staging system. As discussed in a multidisciplinary cancer board, which included experienced hepatobiliary surgeons, hepatologists, and interventional radiologists, surgical resection was recommended first. C-ion RT was recommended as an alternative treatment option, because RFA and SBRT were unsuitable due to the tumor size. Both treatments were proposed to the patient, and the patient opted for C-ion RT.

**Fig. 2:**
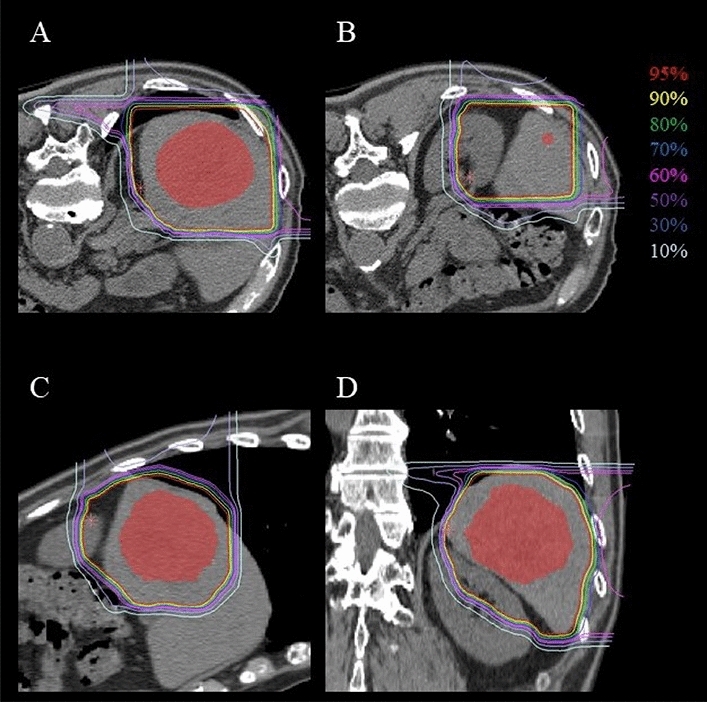
Dose distribution of C-ion RT, where the red-filled area is the gross tumor volume. **A**, **B** Axial images of the primary tumor (**A**) and the satellite nodule (**B**) that are also included in the irradiation field. **C** Sagittal image. **D** Coronal image

The patient underwent simulation and planning using fixation cushions and thermoplastic shells based on respiratory-gated CT. Gross tumor volume (GTV) was contoured based on the fused contrast-enhanced CT, and the clinical target volume (CTV) was enlarged by 5 mm and excluded the organs at risk (OAR; gastrointestinal tract, portal vein). The planning target volume (PTV) was determined using a 3 mm setup margin and internal margin during respiratory gating. The patient then received C-ion RT at a dose of 52.8 Gy (RBE) in 4 fractions for 1 week. The dose distribution of the C-ion RT is shown in Fig. 2. The patient completed C-ion RT with grade 1 radiation dermatitis as an acute toxicity and grade 1 radiation pneumonitis as late toxicity according to the Common Terminology Criteria for Adverse Events (CTCAE) version 4.0.

**Fig. 3:**
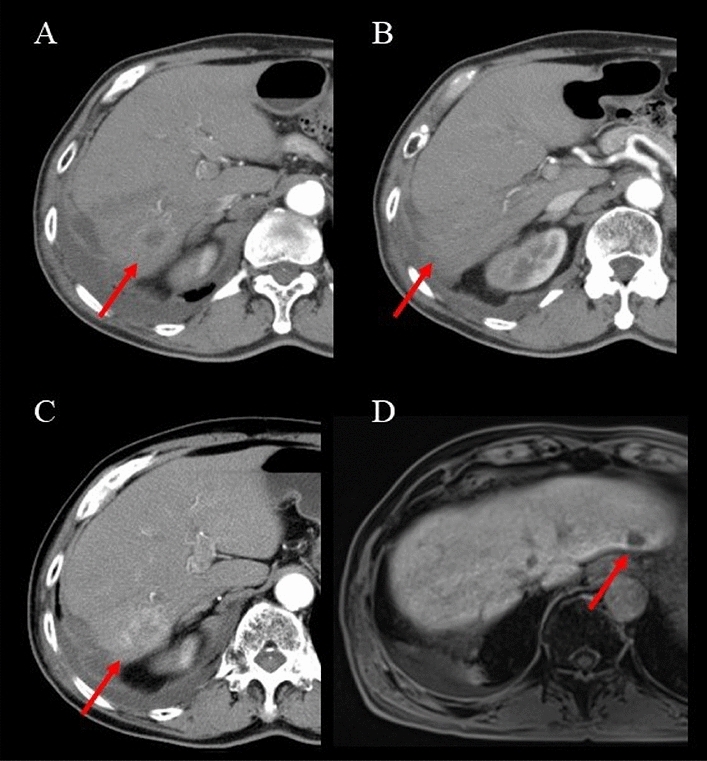
Contrast-enhanced CT and Gd–EOB–DTPA-enhanced MRI after C-ion RT. **A**, **B** Arterial phase CT 12 months after C-ion RT, red arrows show the shrinkage of the primary tumor (**A**) and the disappearance of the satellite nodule (**B**). **C**, **D** Arterial phase CT and hepatocyte phase MRI 24 months after C-ion RT, red arrows show the enhancement of contrast effect of the primary tumor (**C**) and the new lesion in segment 2 (**D**) with contrast defect

At 12 months after C-ion RT, there was no evidence of local recurrence due to tumor shrinkage and the diminished contrast effect, and no distant metastasis was observed. However, 24 months after the initiation of C-ion RT, CT and MRI revealed local recurrence of the primary tumor (segment 6) with an enhancement of contrast effect, with a new lesion in segment 2 of the liver and no evidence of lymph node or distant organ metastasis (Fig. 3). Liver function status at the time of local recurrence was Child–Pugh class A and mALBI grade 1 (Table 1). After discussing with the multidisciplinary board, since the patient did not desire surgical resection or local ablative therapy, TACE used a platinum-based drug (miriplatin, 20–40 mg), and gelatin particles was first administered to the new lesion in segment 2 and the recurrent lesion after C-ion RT.

**Fig. 4:**
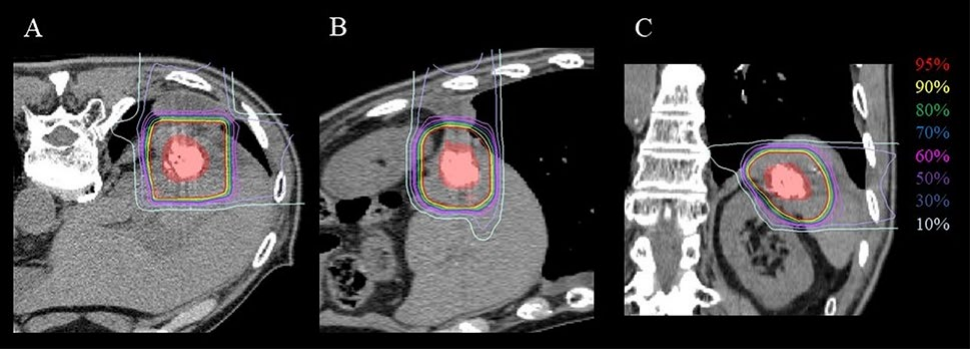
Dose distribution of second C-ion RT, where the red-filled area is the gross tumor volume. **A** Axial image. **B** Sagittal image. **C** Coronal image

Both lesions showed dense lipiodol deposition after TACE, and there was no other lesion. Since the local recurrent lesion was considered refractory to treatment, subsequent C-ion RT was performed at 27 months after initial C-ion RT (3 months after TACE). The administration dose was 60.0 Gy (RBE) in 4 fractions for 1 week. The dose distribution of the C-ion RT is shown in Fig. 4. The patient completed C-ion RT with grade 1 radiation dermatitis as an acute toxicity and grade 1 radiation pneumonitis and rib bone fracture as late toxicity according to CTCAE version 4.0. No adverse events were observed in the hepatobiliary system.

**Fig. 5:**
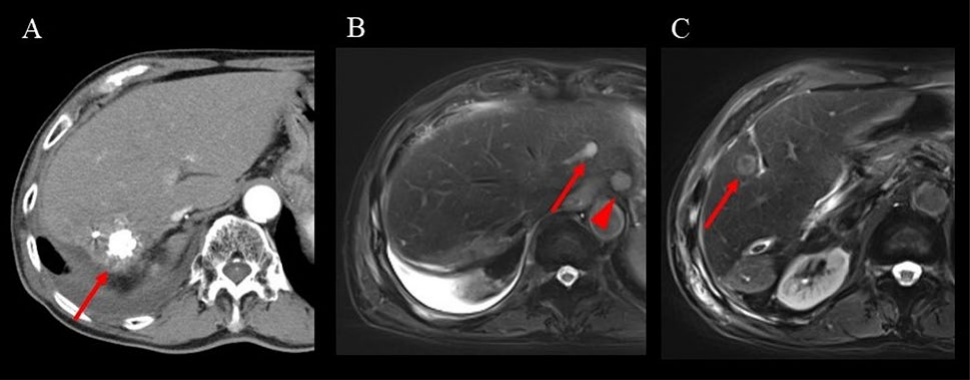
Contrast-enhanced CT after second C-ion RT and superparamagnetic iron oxide (SPIO)-enhanced MRI after all treatments. **A** Arterial phase CT 5 months after second C-ion RT, the red arrow shows the disappearance of the contrast effect. **B**, **C** Hepatocyte phase MRI 60 months after initial C-ion RT, red arrows show intrahepatic lesions in segments 2 and 5. The red triangle shows lymph node metastasis. All lesions were treated with TACE

At 5 months after the second C-ion RT, the contrast effect of HCC in segment 6 of the liver disappeared, and there was no decline in liver function. Subsequently, the patient had repeated intrahepatic recurrences and lymph node metastasis. Systemic therapy using a molecular target agent was suggested; however, he did not desire it due to his age. Since the intrahepatic lesions and metastatic lymph node were fed by a common feeder, he underwent palliative TACE. At 60 months after the initial treatment, the patient did not want any further treatment because of his age. At this time, the patient had multiple intrahepatic lesions and lymph node metastasis (Fig. 5). However, he was independent in daily life, and liver function was well preserved (Table 1). In addition, the patient had been drinking recreationally until just before the final admission. At 88 months after the initial C-ion RT, the patient died of bacterial pneumonia.

## Discussion

We present the case of long-term survival of an elderly patient with HCC treated by a repeated combination of C-ion RT and TACE. Local recurrence after C-ion RT was salvaged by re-irradiation and TACE without deterioration of liver function or severe adverse events.

In elderly patients with HCC, treatment options are limited because of the invasiveness of surgery. Especially in large tumors cases, RFA and SBRT are also not indicated. C-ion RT can be applied to large tumors without complications, including after resection or embolization. In the present report, the patient was discharged immediately after treatment with no severe adverse events and was able to have a similar lifestyle as before two rounds of treatment.

There are no reports of TACE or re-irradiation for local recurrence after C-ion RT. This report suggests the safety of TACE for local recurrence after C-ion RT and the potential for favorable local control with subsequent high-dose C-ion re-irradiation. Oshiro et al. reported that re-irradiation with proton beam therapy is safe and effective for patients, including local recurrence [16], and Su TS et al. reported that SBRT combined with TACE had a higher local effect than SBRT alone [17]. Therefore, re-irradiation with C-ion RT combined with TACE for local recurrence was considered safe and effective. The dose fractions of 52.8 Gy (RBE) and 60 Gy (RBE) in 4 fractions for 1 week for HCC located in the peripheral site were used. In the present case, we used 52.8 Gy (RBE) as a primary treatment and 60.0 Gy (RBE) as a secondary re-irradiation treatment. The dose was increased, because 52.8 Gy (RBE) was considered insufficient for local control because of recurrence and potential radiation resistance after primary C-ion RT. TACE using miriplatin suspended in lipiodol was safe and resulted in dense drug accumulation in the present case. The combined TACE and high-dose administration of 60 Gy (RBE) C-ion RT allowed salvage for local recurrence after initial C-ion RT.

When re-irradiation for HCC is discussed, the dose to the normal liver must be considered, both in terms of high local dose and mean liver dose. There are several reports on the dose to the normal liver after re-irradiation with SBRT. Gkika et al. reported re-irradiation with SBRT for in- and out-of-field recurrences of primary or secondary liver cancer [18]. They reported that the median maximum dose of combined initial and secondary SBRT to the normal liver (Liver-PTV) in patients with in-field recurrence was 359 Gy (EQD_2_, *α*/*β* = 2); however, no adverse events related to liver tolerance were observed in these patients, except for mild ascites controlled with diuretics. Kimura et al. reported re-irradiation with SBRT for out-of-field recurrence of HCC [19]. They reported that the median mean dose of each SBRT to the normal liver (Liver-GTV) was 13.3 Gy, and only biochemical and hematologic toxicities were observed. In the present case, the median maximum dose of combined initial and secondary C-ion RT to the normal liver was 496 Gy (RBE) (EQD_2_, *α*/*β* = 2), and the mean doses of the first and second C-ion RT to the normal liver were 22.1 Gy and 7.8 Gy, respectively. Although both doses were higher than those reported above, which may be due to the prescription dose and tumor size, no severe hepatobiliary toxicities developed. This may be because C-ion RT preserves a large non-irradiated volume of the liver and that the lesion in this case was located in the periphery.

Liver function preservation is crucial for HCC patients who may need repeat treatment because of frequent intrahepatic recurrences. In the present case, the patient needed repeated treatments for multiple recurrent tumors. Our findings suggest that liver function preservation after initial and secondary C-ion RT enables repeated treatment rounds after recurrence. Therefore, C-ion RT offers the advantage of liver function preservation during HCC treatment compared with X-ray radiotherapy, which is based on a dosimetric advantage. TACE combined with C-ion RT may confer a better prognosis than TACE combined with X-ray radiotherapy.

We encountered a case of an elderly patient with a large HCC tumor treated with combined repeated C-ion RT and TACE, resulting in long-term local control and survival. The patient showed liver function preservation after initial and secondary C-ion RT, which enabled multiple treatment rounds after recurrence. High local effects and liver function preservation affected long-term survival, suggesting that C-ion RT might be a minimally invasive treatment option for elderly patients with large HCC tumors and is safe for multiple irradiations of the liver.

## Data Availability

The data sets generated for this study are available upon request to the corresponding author.
